# Use of Bispectrum Analysis to Inspect the Non-Linear Dynamic Characteristics of Beam-Type Structures Containing a Breathing Crack

**DOI:** 10.3390/s21041177

**Published:** 2021-02-07

**Authors:** Li Cui, Hao Xu, Jing Ge, Maosen Cao, Yangmin Xu, Wei Xu, Dragoslav Sumarac

**Affiliations:** 1 College of Mechanics and Materials, Hohai University, Nanjing 210098, China; cuili@hhu.edu.cn (L.C.); xuyangmin12@163.com (Y.X.); wxu@hhu.edu.cn (W.X.); 2 School of Aeronautics and Astronautics, Faculty of Vehicle Engineering and Mechanics, State Key Laboratory of Structural Analysis for Industrial Equipment, Dalian University of Technology, Dalian 116024, China; xuhao@dlut.edu.cn; 3 Jiangsu Hongyuan Science and Technology Engineering Co. Ltd., 801 Changwu Road, Changzhou 213162, China; gejingdgyzmy@hotmail.com; 4 Faculty of Civil Engineering, University of Belgrade, Bulevar Kralja Aleksandra 73, 11000 Belgrade, Serbia; sumi@eunet.rs; 5 College of Civil and Architecture Engineering, Chuzhou University, Chuzhou 239000, China

**Keywords:** bispectrum, breathing crack, finite element analysis, non-linear dynamics, second harmonic

## Abstract

A breathing crack is a typical form of structural damage attributed to long-term dynamic loads acting on engineering structures. Traditional linear damage identification methods suffer from the loss of valuable information when structural responses are essentially non-linear. To deal with this issue, bispectrum analysis is employed to study the non-linear dynamic characteristics of a beam structure containing a breathing crack, from the perspective of numerical simulation and experimental validation. A finite element model of a cantilever beam is built with contact elements to simulate a breathing crack. The effects of crack depth and location, excitation frequency and magnitude, and measurement noise on the non-linear behavior of the beam are studied systematically. The result demonstrates that bispectral analysis can effectively identify non-linear damage in different states with strong noise immunity. Compared with existing methods, the bispectral non-linear analysis can efficiently extract non-linear features of a breathing crack, and it can overcome the limitations of existing linear damage detection methods used for non-linear damage detection. This study’s outcome provides a theoretical basis and a paradigm for damage identification in cracked structures.

## 1. Introduction

Preventing damage from occurring in engineering structures is difficult, due to the long-term effects of loads and environmental factors [1]. A typical example of damage is a crack. This not only affects the integrity of an engineering structure, but also poses a serious threat to its safety and service life [2]. In particular, the presence of cracks in beam structures is found in numerous cases, making basic research into the mechanical behavior of such beams [3] of great significance for diagnosing any structural damage and developing health-monitoring technology.

Traditional damage-detection methods based on signal-processing techniques, typically Fourier transform and power-spectrum analysis, are mostly applied by assuming that engineering structures behave in a linear manner [4]. However, a vast amount of damage that occur in actual engineering structures cause non-linear dynamic behaviors [5,6,7,8]. The non-linear dynamic behaviors are mainly represented by two types: (i) progressive degradation or deterioration of structural properties with time evolution. The yielding of frame structures under seismic action is an example of this type of non-linear damage [9,10,11]; (ii) generation of secondary vibrational resource complicating structural dynamic behavior. This type of non-linear damage is exemplified by breathing cracks [12,13,14] and material stratification [15,16]. Wavelet-based time-frequency methods are representative techniques for characterizing structural progressive degradation or deterioration, the first type of non-linear damage, with its ability to detect the relationship between frequencies and its time dependence, and has a certain noise robustness [17,18,19,20,21,22]. Nevertheless, the method suitable for characterizing the second type of non-linear damage, particularly the breathing crack, is pretty absent while desirable.

The essence of traditional power-spectrum analysis [23], which plays an important role in signal processing, is the analysis of second-order signal statistics. Power-spectrum analysis can only provide information about amplitude-frequency characteristics of signals, but cannot provide high-order statistical information, nor can it effectively suppress the influence of noise. Unlike power-spectrum analysis, bispectral analysis has been explored in applications of non-linear analysis. Collis et al. [24] proved that certain information, unavailable through the examination of second-order statistics such as the spectrum or correlation function, can be obtained by higher-order spectra.

Li et al. [25] applied bispectral analysis for the diagnosis of rotor crack. Yiakopoulos et al. [26] showed that bispectral analysis could effectively extract rolling bearings’ ground-fault characteristics.

In general, bispectrum-based damage diagnosis relies on the correlation between spectral peaks and damage types. Commonly used are the horizontal, diagonal, and central frequency sections of a slice spectrum [27,28,29], and the cepstrum, after the cluster lines are simplified to a single spectral line [30,31].

Moreover, some studies have proved that damage types can be identified according to the spectrum-peak frequency of the bispectrum, and that the degree of damage can be identified by texture features [32]. In existing research and engineering applications, the bispectrum is mostly used in damage detection in rotating machinery such as bearings [33,34], but has rarely been used for damage detection in civil engineering structures. Thus there is a strong incentive to explore the advantages of the bispectrum in non-linear damage detection in civil engineering structures.

For this paper, a finite element model of a beam with a breathing crack was built, using contact elements to simulate the opening and closing characteristics of the breathing crack. Subsequently, the non-linear characteristics of the breathing cracked beam under harmonic load, the influence of excitation frequency and magnitude, crack position and depth, and noise on the non-linear characteristics of the structure were studied systematically.

The results show that the bispectral analysis method demonstrates sensitivity to the non-linear features of the cracked beam, and can effectively overcome the limitations of existing linear damage-detection methods in non-linear damage detection.

## 2. Fundamentals

The effectiveness of traditional damage-detection methods, based on linear hypotheses, is largely limited in non-linear structures. With its capacity to tackle non-linear problems, bispectrum analysis has the lowest order among the high-order spectrum-analysis approaches. Its development is based on the power-spectrum-analysis method. Compared with linear damage-detection methods, bispectrum analysis contains richer damage information and is an effective tool for dealing with non-linear signals.

### 2.1. Definition of Bispectrum

It is assumed that the mean value of a real random signal is zero and its k-order cumulant $c_{kx}(\tau_{1},\tau_{2},\ldots ,\tau_{k-1})$ is stationary. The k-order spectrum is defined as the (*k*-1)-dimensional Fourier transform of $c_{kx}(\tau_{1},\tau_{2},\ldots ,\tau_{k-1})$, expressed as

(1) $$S_{kx}(\omega_{1},\omega_{2}\ldots ,\omega_{k-1})=\sum_{\tau_{1}=-\infty }^{\infty }\ldots \sum_{\tau_{k-1}=-\infty }^{\infty }c_{kx}(\tau_{1},\tau_{2},\ldots ,\tau_{k-1})\cdot \exp\left[-i\sum_{j=1}^{k-1}\omega_{j}\tau_{j}\right]$$

The third-order spectrum, i.e., $S_{3x}$ with *k* = 3, is also called the bispectrum, expressed as $B_{x}\left(\omega_{1},\omega_{2}\right)$. The estimated bispectrum [35,36] can be written as

(2) $$B(\omega_{1},\omega_{2})=X(\omega_{1})X(\omega_{2})X^{*}(\omega_{1}+\omega_{2})$$

where $X(k)=\sum_{n=0}^{N-1}x(n)e^{-i\frac{2\pi }{N}nk}(0\leq k\leq N-1)$ is the Fourier transform determined by the principal value sequence of {x(n)}; ∗ represents the complex conjugate. $B(\omega_{1},\omega_{2})$ is a function with two independent frequencies as the independent variables, i.e., $\omega_{1}$ and $\omega_{2}$, which contain the phase information. Since the bispectrum is symmetric, only the function parts within the regions of $\omega_{1}\leq \omega_{2}$, $0\leq \omega_{2}\leq f_{2}/2$ and $\omega_{1}+2\omega_{2}\leq f_{s}$ need to be calculated, where $f_{s}$ represents the sampling frequency.

In the following study, the bispectrum is treated by logarithm and normalization in order to amplify the harmonic components.

### 2.2. The Features of Bispectral Analysis

#### 2.2.1. Identification of Non-Linear Systems

While the power spectrum represents the distribution of signal energy against frequency, the physical meaning of bispectrum is not explicit. Reference [37] explains the physical meaning of bispectrum: since the secondary moment of zero delay is the variance of the signal, and the third moment of zero delay is the skewness of the signal, the power spectrum is equivalent to the decomposition of signal variance in the frequency domain, whereas the bispectrum is the decomposition of signal skewness in the frequency domain. Therefore, the bispectrum can be used to describe the asymmetric and non-linear characteristics of signals.

#### 2.2.2. Detection of Non-Linear Coupling Features in Signals

Because the bispectrum contains phase information, it can be used to determine whether non-linearity exists in the signal by detecting the quadratic phase coupling. Assume there is a sine wave $x(n)=\cos(k_{0}n)$, with the period of $N=\frac{2\pi }{k_{0}}$. The Fourier transform determined by the principal value sequence is:

(3) $$X(k)=\sum_{n=0}^{N-1}x(n)e^{-i\frac{2\pi }{N}nk}=\frac{1}{2}\sum_{n=0}^{N-1}\left[e^{-ik_{0}n(k-1)}+e^{-ik_{0}n(k+1)}\right]=\frac{N}{2}[\delta (k-1)+\delta (k+1)]$$

where k=±1, X(k)≠0. The bispectrum is estimated as $B_{x}(k_{1},k_{2})=X(k_{1})X(k_{2})X^{*}(k_{1}+k_{2})$. Since $k_{1}=\pm 1$, $k_{2}=\pm 1$ and $k_{1}+k_{2}=\pm 1$ cannot hold at the same time, $B_{x}(k_{1},k_{2})$ is equal to zero. Assume there is a sinusoidal signal with the harmonic $y(n)=\cos(k_{0}n)+\cos(2k_{0}n)$, with a period of $N=\frac{2\pi }{k_{0}}$. The Fourier transform determined by the principal value sequence is:

(4) $$Y(k)=\sum_{n=0}^{N-1}y(n)e^{-i\frac{2\pi }{N}nk}=\frac{N}{2}[\delta (k-1)+\delta (k+1)+\delta (k-2)+\delta (k+2)]$$

where k=±1 or k=±2, Y(k)≠0. The bispectrum is estimated as $B_{y}(k_{1},k_{2})=Y(k_{1})Y(k_{2})Y^{*}(k_{1}+k_{2})$. Obviously, $B_{y}(k_{1},k_{2})$ has six non-zeros $\left(k_{1}=1,k_{2}=-2\right)$, $\left(k_{1}=1,k_{2}=1\right)$, $\left(k_{1}=-1,k_{2}=-1\right)$, $\left(k_{1}=-1,k_{2}=2\right)$, $\left(k_{1}=-2,k_{2}=1\right)$, $\left(k_{1}=2,k_{2}=-1\right)$. Compared with the case without a harmonic signal, the bispectrum of y(n) is coupled at 6 points. It can be concluded that the frequency coupling features can be obtained by the bispectrum in the analysis of non-linear signals.

#### 2.2.3. Immunity to Gaussian Noise

Assume there are two vibration signals, x(n)=e(n), y(n)=s(n)+e(n), where s(n) is the basic signal containing the second harmonic and e(n) is Gaussian noise. The traditional power-spectrum estimation method is used to analyze the two vibration signals,

(5) $$P_{x}(\omega )=P_{e}(\omega ),$$

(6) $$P_{y}(\omega )=P_{s}(\omega )+P_{e}(\omega )$$

Obviously, the ability of power-spectrum estimation to detect the second harmonic is related to the signal-to-noise ratio (SNR). The lower the SNR, the lower the accuracy of the second harmonic will be. If bispectral analysis is used, i.e.,

(7) $$B_{x}(\omega_{1},\omega_{2})=B_{e}(\omega_{1},\omega_{2}),$$

(8) $$B_{y}(\omega_{1},\omega_{2})=B_{s}(\omega_{1},\omega_{2})+B_{e}(\omega_{1},\omega_{2})$$

In general, if the probability density function of noise is approximately symmetric about the longitudinal axis, $B_{e}(\omega_{1},\omega_{2})$ can be ignored, so then the Equations (7) and (8) become:

(9) $$B_{x}(\omega_{1},\omega_{2})=0,$$

(10) $$B_{y}(\omega_{1},\omega_{2})=B_{s}(\omega_{1},\omega_{2})$$

It can be seen that, even in the condition of low SNR, the SNR can be improved by bispectrum analysis, which is more conducive to the detection of non-linear signals. Zhou et al. [38] showed that the bispectrum analysis method could obtain more effective detection than the traditional Fourier transform method in the case of low SNR for signals mixed with random or Gaussian noise.

### 2.3. Calculation of the Bispectrum

The methods of high-order spectrum estimation can be divided into two categories: parametric and non-parametric. The advantages of non-parametric high-order spectrum estimation include ease of implementation and availability of the Fourier transformation (FFT). Moreover, non-parametric algorithms can be divided into direct and indirect methods [29]. For this paper, direct non-parametric bispectrum estimation is used.

Assuming that the random signal {x(n)} has N sample points, i.e., x(1), x(2), …, x(N), the implementation steps are as follows:

(1) Sample segmentation:

The N-point data is divided into K segments. Each segment contains M sample values, i.e., N=KM. M should meet the general length requirement of FFT. The data in the j^th^ segment are recorded as $x^{\left(j\right)}(n)$, j=1,2,…,K. If needed, zeros can be added at the end of each segment to meet the length requirement. As with power spectrum estimation, the segments can overlap to meet the requirements of frequency resolution and estimation variance.

(2) Calculation of discrete Fourier coefficients:

(11) $$Y^{j}(\lambda )=\frac{1}{M}\sum_{n=0}^{M-1}x^{j}(n)e^{-j\frac{2\pi }{M}n\lambda }$$

where, $\lambda =0,1,\ldots ,\frac{M}{2}-1$.

(3) Calculation of the third order cumulant:

(12) $${\hat{b}}^{j}\left(\lambda_{1},\lambda_{2}\right)=\frac{1}{\Delta_{0}^{2}}\overset{L_{1}}{\underset{k_{1}=-L_{1}}{\sum }}\overset{L_{2}}{\underset{k_{2}=-L_{2}}{\sum }}Y^{j}\left(\lambda_{1}+k_{1}\right)Y^{j}\left(\lambda_{2}+k_{2}\right)Y^{j}{}^{*}\left(\lambda_{1}+\lambda_{2}+k_{1}+k_{2}\right)$$

where, $\Delta_{0}=\frac{f_{s}}{N_{0}}$.

(4) Calculation of the bispectrum average:

(13) $$B_{x}\left(\lambda_{1},\lambda_{2}\right)=\frac{1}{K}\overset{K}{\underset{i=1}{\sum }}{\hat{b}}^{j}\left(\lambda_{1},\lambda_{2}\right)$$

A contradiction between estimation variance and frequency resolution has frequently been encountered in traditional spectrum estimation methods. In practical applications, the final frequency resolution,$\frac{f_{s}}{N}$, is selected on the premise of balanced estimation variance and frequency resolution.

## 3. Numerical Simulation

In this section, the finite element (FE) method was used to study the non-linear dynamic characteristics of a beam with a breathing crack.

### 3.1. Finite Element (FE) Modeling of a Cantilever Beam with a Breathing Crack

**Figure 1:**
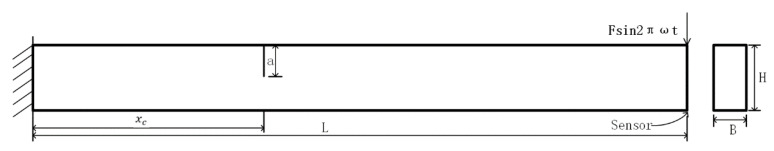
Geometry sketch of a cantilever beam with a breathing crack.

As illustrated in Figure 1, a three-dimensional, rectangular section cantilever beam was built using FE software ANSYS with solid45 elements which are defined by eight nodes having three degrees of freedom at each node. The geometric parameters of the beam, including the length (*L*), width (*B*), height (*H*), and the material parameters, including elastic modulus (*E*), Poisson’s ratio (*ν*), and density (*ρ*), are shown in Table 1. The cantilever beam contained a unilateral penetrating breathing crack, with the depth of *a* and the distance from the fixed end of $x_{c}$. A harmonic excitation Fsin2πωt was applied on the upper surface near the free end of the beam, and a sensing point was arranged on the lower surface near the free end to trace and record the dynamic responses of the beam. In the model, there were a total of 5120 elements, and the elements near the crack are refined to precisely capture the dynamic behaviors of the crack. The depth and position of the crack were signified by the dimensionless parameters p=a/H and $q=x_{c}/L$, respectively.

For simplicity, an open crack model is often used to ignore the non-linear characteristics induced by cracks. However, the open crack model is not consistent with actual cases, and studies have shown that crack closure should be taken into account for more accurate representation of structural responses. Gudmundson et al. [7] pointed out that the change of structural natural frequency, due to open cracks, is much smaller than those due to breathing cracks. Moreover, the open-crack model may underestimate the severity of a fatigue crack, resulting in increased risks to safety. Qian et al. [8] found that the difference in the displacement responses between intact and cracked beams decreases when an open-close crack model is used.

**Figure 2:**
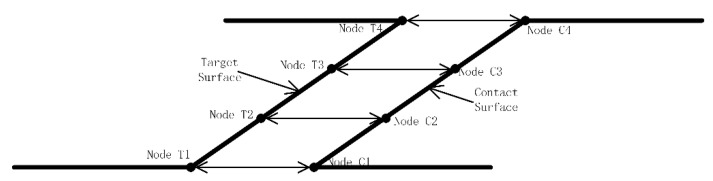
Schematic diagram of face-to-face contact model.

In this work, determining the opening and closing behaviors of the breathing crack was considered a local contact problem. There are three common contact algorithms used by ANSYS, this time using the generating function algorithm. Taking one crack surface as the target surface and the other as the contact surface, the contact behaviors of cracks were studied using the surface-to-surface contact method, as illustrated in Figure 2. Specifically, the target surface was allowed to penetrate the contact surface, but the contact surface was not allowed to penetrate the target face. During the vibration of the cantilever beam, there were three states of the breathing crack: (a) the crack was completely open, and no nodes on the target and contact surfaces were in contact; (b) the crack was completely closed, and all nodes on the target and contact crack surfaces were in contact; (c) in the transition state featuring pressure release or pressure accumulation, the nodes on the target and contact crack surfaces were in partial contact.

### 3.2. Non-Linear Dynamic Characteristics of the Cantilever Beam Containing a Breathing Crack

**Figure 3:**
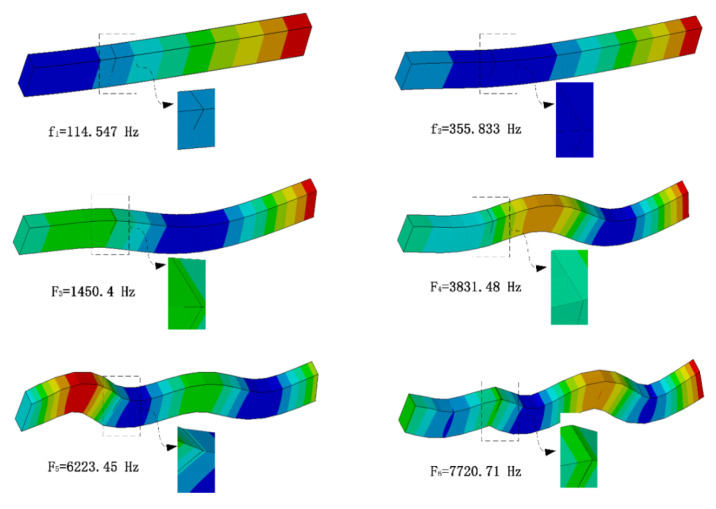
The first six modal frequencies and modes when *p* = 50% and q = 30%.

The dynamic characteristics of the cracked-beam structure, including modal frequencies and mode shapes, were obtained by modal analysis. Figure 3, below, shows the first six modal frequencies and mode shapes of the beam with *p* = 50% and q = 30%. It shows that the cracks in modes 1, 4, and 5 were in a closed state. In mode 4, in particular, the target surface penetrated the contact surface. The crack in modes 2, 3, and 6 was open, where modes 2 and 3 correspond to the transition state, and it is difficult to judge whether mode 6 was in transition or in a fully opened state.

To diagnose damage in rotating machinery, the use of harmonics as typical signs of non-linearity of structural responses has been common, not only in research but also in engineering practice. However, structural non-linear characteristics such as harmonics caused by a breathing crack are difficult to identify using a linear damage-detection method.

Hence, bispectral analysis was employed to identify the relationships between the harmonic components in the signal, which could be used to identify the harmonic coupling in the response and to estimate the degree of non-linearity.

A harmonic excitation of $\omega =\frac{1}{2}f_{1}$, F = 500 N was applied at the position shown above in Figure 1. A dynamic time-history calculation of the structure was carried out, the step size was 5 × $10^{-4}$ s, and the total analysis time was 2.548 s. The acceleration response at the sensing point was extracted and bispectral analysis performed.

**Figure 4:**
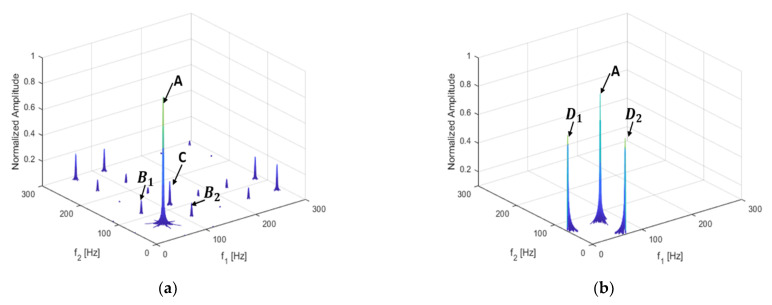
Bispectrum of the cantilever structure (**a**) when *p* = 50%, q = 30%; (**b**) when there is no damage.

The result of the damaged beam is shown below in Figure 4a; Figure 4b shows the bispectrum of the beam without damage. Figure 4b shows only the main peak A, caused by the excitation frequency coupling at (57 Hz, 57 Hz), and peaks $D_{1}$ and $D_{2}$, excited by the load without coupling at (57 Hz, 0 Hz) and (0 Hz, 57 Hz).

Compared with Figure 4b, several harmonics can be seen in Figure 4a in terms of the coupling subpeaks of the excitation frequency and the second harmonic $B_{1}$ and $B_{2}$ at (57 Hz, 114 Hz) and (114 Hz, 57 Hz), and the self-coupling subpeak of the second harmonic C at (114 Hz, 114 Hz), along with other harmonic components.

This shows that harmonic and frequency coupling can be used to identify non-linear damage and to provide damage-identification indicators for the development of a structural health diagnosis and crack identification methods. By using bispectral analysis, not only can the harmonics of each order, caused by non-linearity, be observed, but also the coupling relations among frequencies.

**Figure 5:**
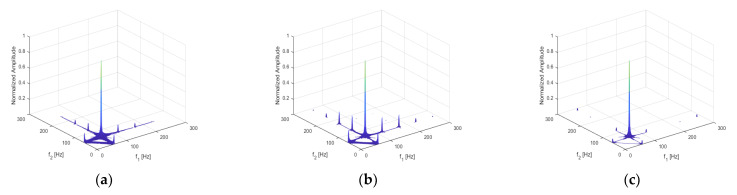
Bispectrum of (**a**) displacement response; (**b**) velocity response; (**c**) stress response.

In addition to the acceleration response, the bispectrum can also analyze displacement, velocity, and stress responses. The corresponding results are shown below in Figure 5a–c, respectively. In the figures, the second-order harmonics can be observed, although their amplitudes exhibit clear deviations.

### 3.3. The Influence of Crack Depth on Non-Linear Dynamic Features

Because crack propagation is unavoidable during structural service life, crack-depth identification is critical. For this reason, understanding the relationship between non-linear dynamic characteristics and crack-depth variation is crucial.

We assumed that the crack was located at q = 0.1, but the crack depth was set to *p* = 0.1, 0.2, 0.3, 0.4, and 0.5. The excitation frequency was $\omega =\frac{1}{2}f_{1}$. As explained in [39], a 1/2 super harmonic resonance was expected to occur at this frequency, and here, frequency components were easily fully generated. The magnitude of the excitation was F = 500 N.

**Figure 6:**
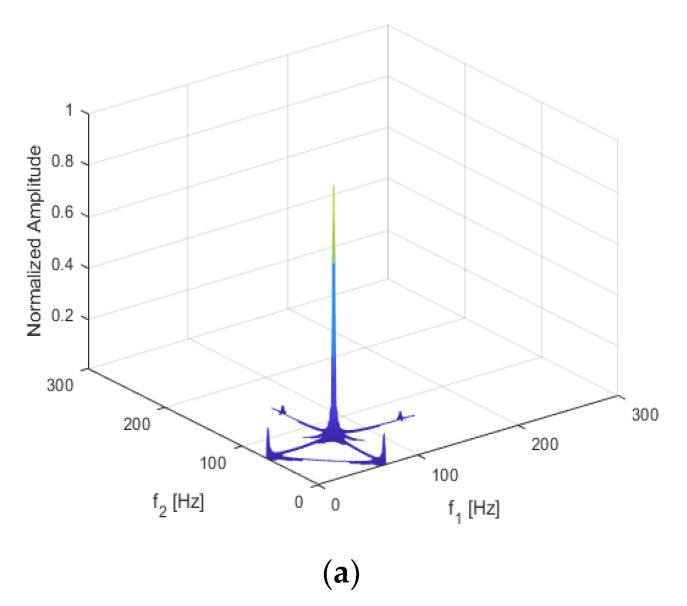

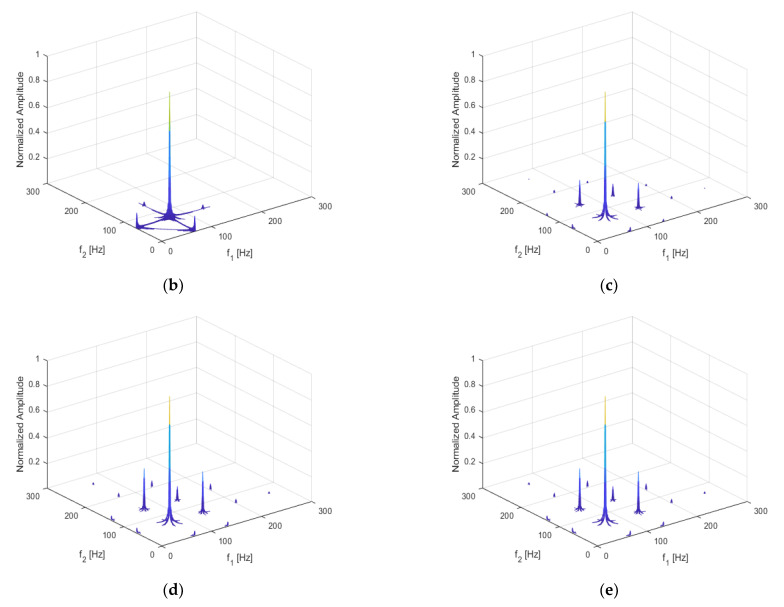
Bispectrum when (**a**) *p* = 0.1; (**b**) *p* = 0.2; (**c**) *p* = 0.3; (**d**) *p* = 0.4; (**e**) *p* = 0.5.

**Figure 7:**
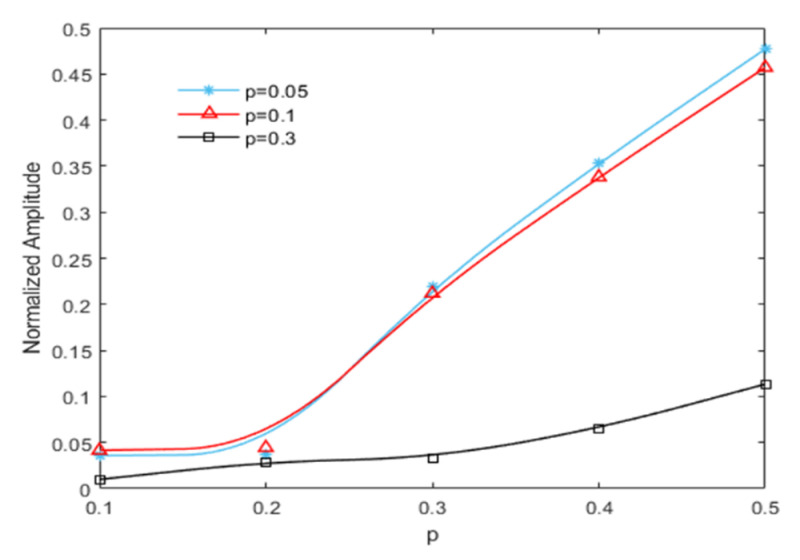
Variation trend of the second harmonic amplitude with crack depth.

The acceleration responses were extracted for bispectral analysis, giving rise to three-dimensional bispectral images, as shown in Figure 6. The amplitudes of the second harmonic at each crack depth were extracted, with the variation trends shown below in Figure 7. It was apparent that the amplitude of the second harmonic increased monotonically in proportion to the increase in crack depth. Therefore, the amplitude of the second harmonic in the bispectrum can be used as a damage-sensitive feature to indicate the degree of damage of the cracked beam.

### 3.4. The Influence of Crack Location on Non-Linear Dynamic Features

**Figure 8:**
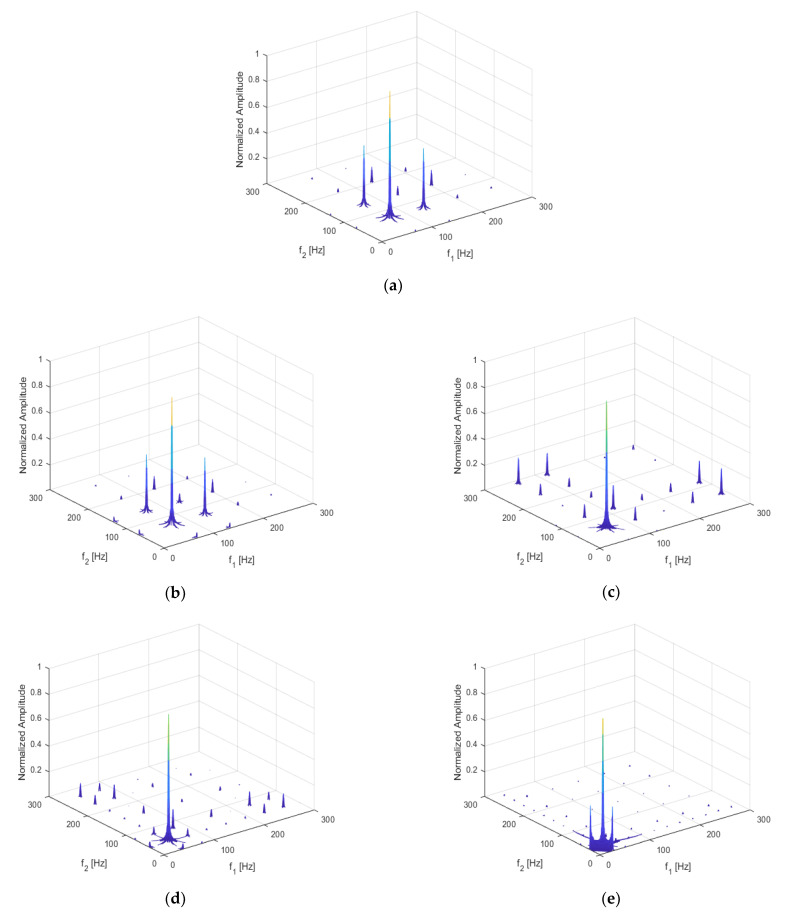
Bispectrum when (**a**) q = 5%; (**b**) q = 10%; (**c**) q = 30%; (**d**) q = 50%; (**e**) q = 70%.

**Figure 9:**
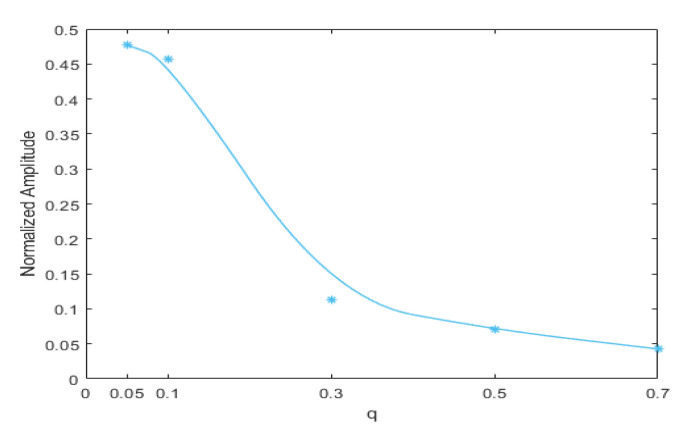
Variation trend of the second harmonic amplitude with crack position.

For the beam with a breathing crack, as studied for this paper, the local stress field in the beam changed with the position of the damage, which in turn changed the non-linear dynamic features of the structure. Assuming a fixed crack depth of *p* = 0.5, the crack location was set to q = 0.05, 0.1, 0.3, 0.5, and 0.7, respectively. The excitation frequency and magnitude were kept at $\omega =\frac{1}{2}f_{1}$ and F = 500 N, respectively. The three-dimensional bispectral images obtained are shown above in Figure 8a–e and Figure 9, below, shows the variation trend of the second harmonic amplitude with the crack position.

It can be seen that the amplitude of the second-order harmonic decreased monotonically when the crack moved from the fixed end to the free end. When q = 0.7, the harmonics could still be observed, but the amplitudes became minimal.

This result indicates that the closer the crack location is to the fixed end, the more significant is the structural non-linearity.

### 3.5. The Influence of Harmonic Excitation Frequency on Non-Linear Dynamic Features

**Figure 10:**
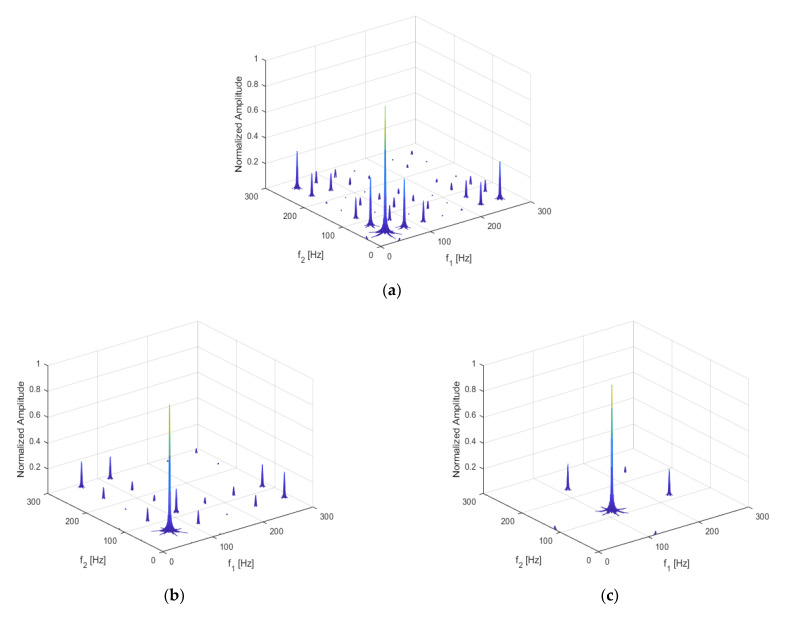

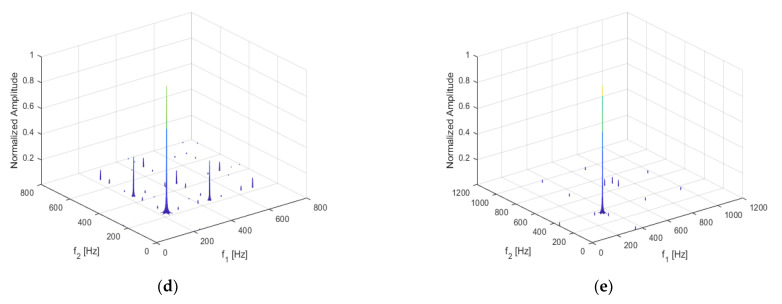
Bispectrum when (**a**) $\omega =\frac{1}{3}f_{1}$; (**b**) $\omega =\frac{1}{2}f_{1}$; (**c**) $\omega =f_{1}$; (**d**) $\omega =2f_{1}$; (**e**) $\omega =3f_{1}$.

To better study the non-linearity caused by the periodic movement of the breathing crack, the excitation frequency was adjusted to be $\omega =\frac{1}{3}f_{1}$, $\frac{1}{2}f_{1}$, $f_{1}$, $2f_{1}$, and $3f_{1}$, to investigate the variation features of non-linear dynamic characteristics. The crack location and depth were kept at q = 0.3 and *p* = 0.5, respectively. Acceleration responses were extracted, and bispectral analysis was then performed to obtain the three-dimensional bispectrum images, shown below in Figure 10.

It can be seen that, when the structure was subjected to harmonic excitation, multiple harmonic components in the response could be analyzed by the bispectral method, including the coupling of excitation frequencies, n-order harmonic components, coupling of n-order harmonic components and m-order harmonic components.

The coupling of excitation frequencies and the second harmonic was obvious in each bispectral image. When the excitation frequency was an integer times (*n* = 1, 2, 3) or one over an integer times (*n* = 1/3, 1/2) the first-order frequency of the structure, the harmonic components of the cracked beam could be fully excited. Frequency coupling at $\omega =\frac{1}{3}f_{1}$, $\frac{1}{2}f_{1}$, $f_{1}$, and $2f_{1}$, was significant. Specifically, the coupling effect at $\omega =\frac{1}{3}f_{1}$ was more complex, whereas $\omega =f_{1}$ may have induced too much uncertainty to the structural vibration. Therefore, $\omega =\frac{1}{2}f_{1}$ and $\omega =2f_{1}$ could be chosen as the optimal excitation frequencies.

### 3.6. The Influence of Harmonic Excitation Magnitude on Non-Linear Dynamic Features

Another important parameter likely to cause differences in bispectral analysis results was the magnitude of excitation. Assuming that the crack location and depth were q = 0.3 and *p* = 0.5, respectively, and the excitation frequency was $\omega =\frac{1}{2}f_{1}$, six excitation amplitudes, of F = 50 N, 100 N, 250 N, 400 N, 750 N and 1000 N, respectively, were studied.

**Figure 11:**
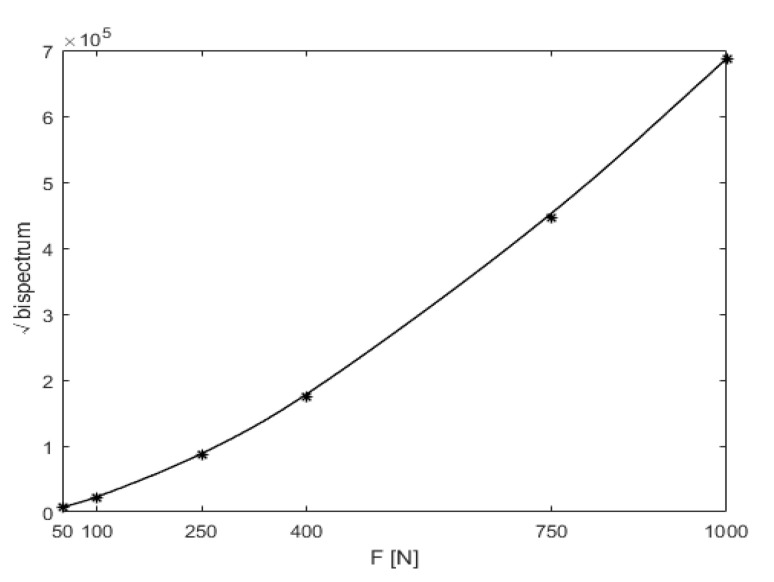
Variation trend of the second harmonic amplitude with the harmonic excitation magnitude.

The bispectrum analysis shows results similar to those above in Figure 10b, and the variation trend of the second harmonic amplitude with the harmonic excitation magnitude is shown below in Figure 11. In particular, in order to study the influence of harmonic excitation magnitude on non-linear dynamic features, the amplitudes of the second harmonic were not normalized.

It was evident that when the excitation magnitude was magnified by 20 times, from 50 N to 1000 N, the non-linear characteristics of the bispectrum, the amplitude of the second harmonic also increased.

### 3.7. The Influence of Noise on Non-Linear Dynamic Characteristics

In actual measurement scenarios, noise from different environmental sources (such as thermal noise, mechanical relaxation, and electrical noise from the data-acquisition system [40]) inevitably affects the measured response data. Noise is likely to affect the representation of non-linear features, and can even drown out the non-linear features of the response.

V.A. Krysko-Jr. concluded in [41] that with the increase in the external transverse load, even a relatively small intensity of white noise has an essential influence on the character of structural vibrations. Theoretically, the bispectrum analysis method used for this paper was immune to Gaussian noise. This section aims to verify the immunity of bispectrum analysis to the influence of noise.

If the crack location and depth are q = 0.3 and *p* = 0.5, respectively, the excitation frequency and magnitude would be $\omega =\frac{1}{2}f_{1}$ and F = 500 N, respectively. Gaussian white noise was introduced into the acceleration response to simulate the effect of environmental noise. The noise intensity was characterized by the SNR, defined as

(14) $$SNR=10\mathrm{lg}\frac{P_{s}}{P_{n}}$$

where $P_{s}$ represents the effective power of the signal and $P_{n}$ represents the effective power of the noise.

**Figure 12:**
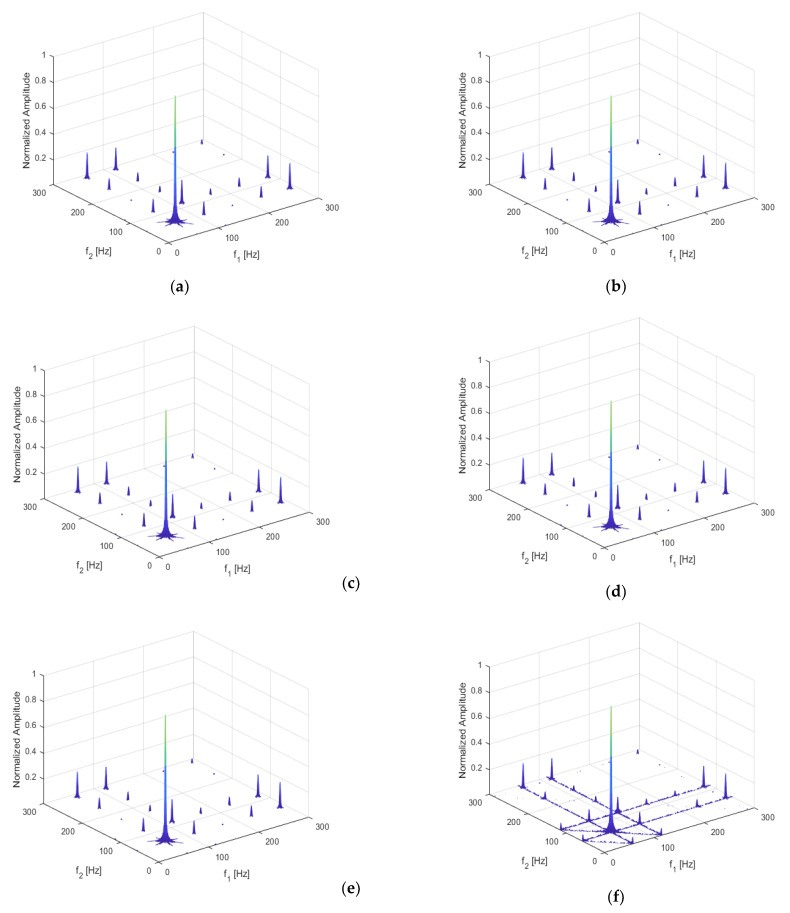
Bispectrum when (**a**) the signal is noiseless; (**b**) SNR = 90 dB; (**c**) SNR = 70 dB; (**d**) SNR = 50 dB; (**e**) SNR = 30 dB; (**f**) SNR = 10 dB.

**Figure 13:**
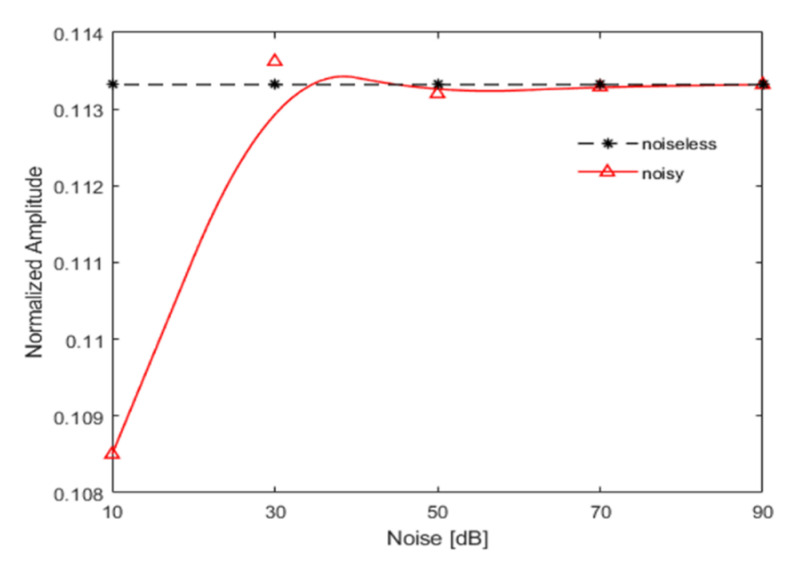
Variation trend of the second harmonic amplitude with signal-to-noise ratio (SNR).

The acceleration responses were extracted and bispectral analysis was used to obtain three-dimensional bispectral images, as shown below in Figure 12. It can be clearly seen that when the SNR is greater than 30 dB, there is no significant change in the bispectrum. However, when the SNR reaches 10 dB, disturbances caused by Gaussian white noise begin to appear. The amplitudes of the second harmonic, under different noise levels, are shown below in Figure 13. It is clear that although the amplitude of the second harmonic of the bispectrum fluctuates around the noiseless signal due to the influence of noise, the characterization of non-linear dynamic features was not seriously impaired, even under the SNR of 10 dB. Such observation indicates that bispectral analysis has strong noise resistance in processing non-linear signals.

## 4. Experimental Validation

In this section, two experiments were carried out to investigate the effects of crack and excitation parameters on the non-linear dynamic features of the cantilever beam with a breathing crack.

### 4.1. Experiment 1

**Figure 14:**
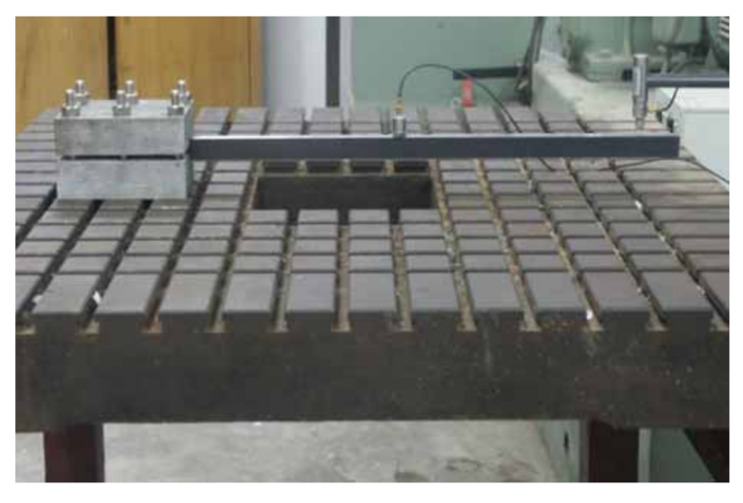
Tested cantilever beam.

A cantilever beam with constant rectangular section, as shown below in Figure 14, was made with a length of L = 0.6 m, cross-section dimensions H × B = 0.019 m × 0.012 m, material density 7750 $\mathrm{kg}/m^{3}$, elastic modulus E = $1.84\times 10^{11}$ Pa, and Poisson’s ratio ν = 0.3.

An accelerometer was mounted on the beam’s surface 90 mm from the free end, and hammer excitation was applied 45 mm from the free end. The acceleration response was amplified by a charge amplifier and then collected by the UTel data-acquisition system at a sampling frequency of 12,800 Hz.

This experiment mainly investigated the influence of crack parameters on the non-linear dynamic features of the cracked beam. The crack parameters are shown in Table 2.

The acceleration responses under different crack parameters were extracted, and the amplitude of the second harmonic components with different crack depths and positions were analyzed using the bispectrum.

**Figure 15:**
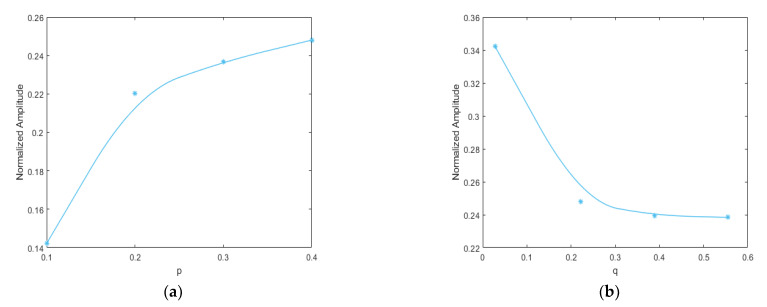
Variation trends of the second harmonic amplitude with (**a**) crack depth; (**b**) crack location.

Figure 15a,b show the variation trend of the second harmonic amplitude with the crack depth and location, respectively. As with the numerical results, the amplitude of the second harmonic of the bispectrum increased along with the increase in crack depth. On the other hand, as the crack moved closer to the fixed end, the amplitude of the second harmonic of the bispectrum increased continuously.

### 4.2. Experiment 2

A steel cantilever beam with the dimensions 380 mm × 20 mm × 10 mm was made, having the material elastic modulus of 193 GPa and Poisson’s ratio of 0.29. The dynamic displacement response was measured in the experiment. To generate crack breathing in the beam, a v-shaped notch 1 mm in depth was first created on one side of the beam, 308 mm from the free end. Cyclic load was then applied to extend the crack depth to 5 mm.

**Figure 16:**
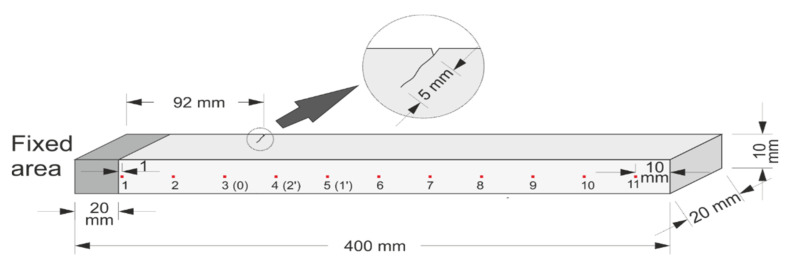
Fatigued steel cantilever beam draft.

Ranging from the fixed end to 4 mm from the free end, 11 measurement points were uniformly arranged along the centerline of the beam, as shown above in Figure 16. An excitation was applied 10 mm from the fixed end. Natural frequencies of different orders of the structure were captured within the ranges 0–2 kHz, 2–20 kHz, and 20–50 kHz, respectively. The first three orders of the natural frequencies measured $f_{1}$ = 70.63 Hz, $f_{2}$ = 688.70 Hz, and $f_{3}$ = 1787.19 Hz, respectively.

This experiment mainly investigated the influence of excitation parameters on the non-linear dynamic features of the cracked beam. The excitation parameters are shown above in Table 3. The acceleration responses under different excitation parameters were extracted, and the amplitude of the second harmonic varying with the excitation frequency and magnitude were subjected to bispectral analysis. A laser-scanning vibrometer, Polytec PSV-400, was used for non-contact measurement of the displacements. PZT piezoceramics were used to excite the structure, and the magnitude of the excitation was proportional to the output amplitude of the piezoceramics generator. Thus, changes in the magnitude of excitation were achieved by changing the voltage.

**Figure 17:**
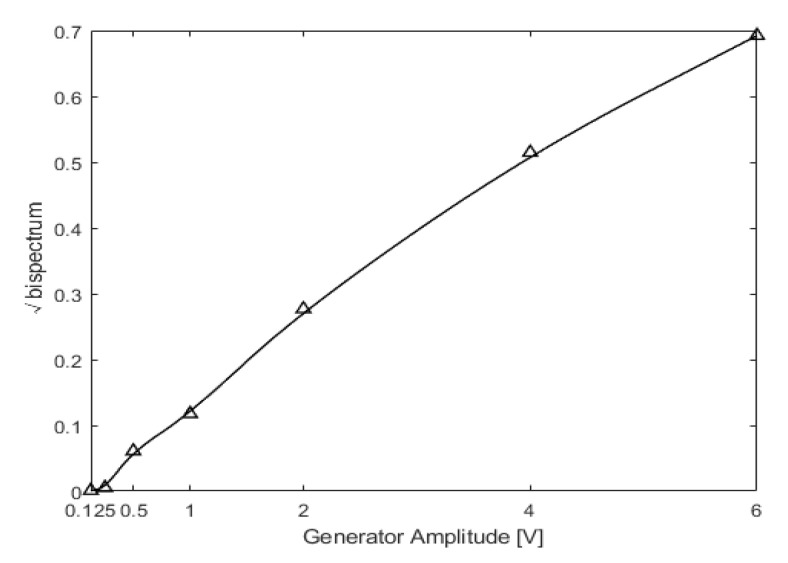
Variation trend of the second harmonic amplitude with the voltage.

Figure 17 below shows the amplitude of the second harmonic varying with voltage, which was directly correlated with the magnitude of excitation. The experimental results were similar to those of the numerical simulation. When the voltage was magnified by 48 times, from 0.125 V to 6 V, the amplitude of the second harmonic also increased.

**Figure 18:**
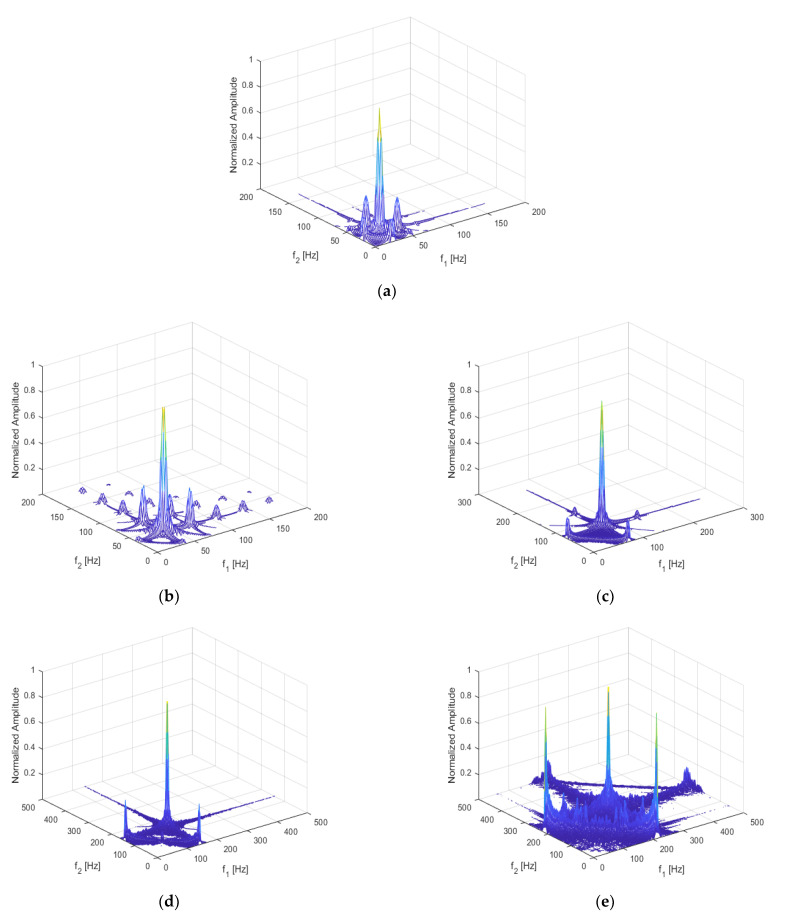
Bispectrum when (**a**) $\omega =\frac{1}{3}f_{1}$; (**b**) $\omega =\frac{1}{2}f_{1}$; (**c**) $\omega =f_{1}$; (**d**) $\omega =2f_{1}$; (**e**) $\omega =3f_{1}$.

Figure 18 shows the bispectrum under different excitation frequencies. In the figure, the second harmonic of the bispectrum under different excitation frequencies can be observed. When the frequency is one over an integer times of the first-order frequency of the structure (e.g., *n* = 1/3, 1/2), the non-linearity can be characterized well and the coupling of other frequencies can be observed. However, when the harmonic excitation frequency is an integer times the first-order frequency (e.g., *n* = 1, 2, 3), the second harmonic is not obvious, especially under the frequency of $3f_{1}$, where the frequency coupling is more complex. Therefore, $\omega =1/2f_{1}$ is expected to generate both obvious and explicit frequency coupling phenomena.

**Figure 19:**
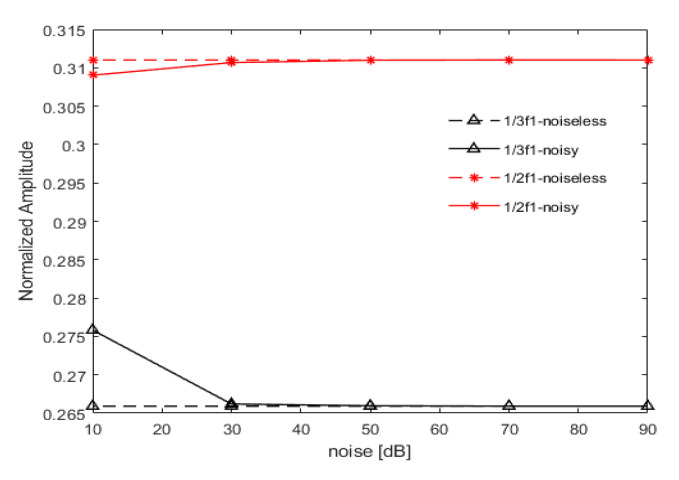
Variation trend of the second harmonic amplitude with noise.

Noise immunity was studied with the excitation frequencies of $\frac{1}{3}f_{1}$ and $\frac{1}{2}f_{1}$ and an excitation voltage of 6 V. As observed in the numerical results, the bispectral analysis showed a certain immunity to Gaussian noise. As shown in Figure 19 with the noise levels of 90, 70, 50, and 30 dB, the Gaussian noise had almost no effect on the amplitude of the second bispectral harmonic. When the SNR reached 10 dB, the amplitude of the second harmonic showed relatively large fluctuations, but the actual errors were only 3.73% and 0.637%, respectively. When the excitation frequency was $\frac{1}{2}f_{1}$, the error was smaller, and when the excitation frequency was $\frac{1}{3}f_{1}$, the error was slightly larger.

However, the magnitude of error did not affect the non-linear characterization of the bispectrum. The immunity of bispectrum analysis to noise influence is demonstrated well.

## 5. Conclusions

For this paper, the non-linear dynamic characteristics of a cantilever beam containing a breathing crack were studied using bispectral analysis. Numerical simulation and experimental verification show that, compared with traditional damage identification techniques, the bispectral analysis method demonstrates sensitivity to the non-linear features of the cracked beam and can effectively overcome the limitations of existing linear damage detection methods in non-linear damage detection. Several conclusions can be drawn:

(1) The bispectral analysis method can not only characterize the non-linear features of crack structures, but can also reveal the coupling relationships among frequencies.
(2) The non-linear feature of the bispectrum, in terms of the amplitude of the second harmonic, is proportional to crack depth, so the amplitude of the second harmonic of the bispectrum can be used as a damage-sensitive feature to quantify the severity of cracks. With the crack position effect, the closer the crack is to the fixed end, the larger is the amplitude of the bispectral second harmonic, and the more obvious are the non-linear features.
(3) As demonstrated in the numerical simulation and experimental results, the harmonic components in the dynamic response signal are the most abundant, and the non-linear characteristic effect is best when the excitation frequency ω is $\frac{1}{2}f_{1}$. The amplitude of the second harmonic increases with the increase of the excitation.
(4) The bispectral analysis method has satisfactory noise robustness. Even under the SNR of 10 dB, the non-linear dynamic characterization of the bispectrum is not affected.

## Figures and Tables

**Table 1:** Parameters of the cantilever beam model.

*L*	*B*	*H*	*E*	*ν*	ρ
(mm)	(mm)	(mm)	(GPa)	1	($\mathrm{kg}/m^{3}$)
500	20	40	70	0.3	2700

**Table 2:** Crack parameters of the cantilever beam with a breathing crack.

Group	Crack Location q (From the Fixed End)	Crack Depth *p*
1	133.3 mm	10%
		20%
		30%
		40%
2	16.7 mm	40%
	133.3 mm	
	233.3 mm	
	333.3 mm	

**Table 3:** Harmonic excitation parameters.

Group	Excitation Frequency (Hz)	Voltage (v)
1	1/2$f_{1}$	0.125
		0.25
		0.5
		1
		2
		4
		6
2	1/3$f_{1}$	6
	1/2$f_{1}$	
	$f_{1}$	
	2$f_{1}$	
	3$f_{1}$	

## Data Availability

Data sharing not applicable No new data were created or analyzed in this study. Data sharing is not applicable to this article.
